# Safety monitoring of artemisinin combination therapy through a national pharmacovigilance system in an endemic malaria setting

**DOI:** 10.1186/1475-2875-12-54

**Published:** 2013-02-05

**Authors:** Sylla Thiam, Jean-Louis Ndiaye, Ibrahima Diallo, Patrick Gatonga, Fatou Ba Fall, Ndella E Diallo, Babacar Faye, Mamadou L Diouf, Medoune Ndiop, Mame B Diouf, Oumar Gaye, Moussa Thior

**Affiliations:** 1 African Medical and Research Foundation, Nairobi, Kenya; 2 Department of Parasitology, Medical Faculty, Université Cheikh Anta Diop, Dakar, Senegal; 3 National Malaria Control Programme, Dakar, Senegal

**Keywords:** Pharmacovigilance, Malaria, ACT, Senegal

## Abstract

**Background:**

The National Malaria Control Programme in Senegal, introduced since 2006, artemisinin-based combination therapy (ACT administration) for the treatment of uncomplicated malaria cases. In this framework, an anti-malarial pharmacovigilance plan was developed and implemented in all public health services. This study investigated the occurrence of Adverse Drug Events (ADEs) after ACT.

**Methods:**

The study was conducted between January 2007 and December 2009. It was based on spontaneous reports of ADEs in public health facilities. Data on patient demographic characteristics, dispensing facility, adverse signs and symptoms and causality were collected from a total of 123 patients.

**Results:**

The age range of these patients was six months to 93 years with a mean of 25.9 years. Of the reported symptoms, 46.7% were related to the abdomen and the digestive system. Symptoms related to the nervous system, skin and subcutaneous tissue, circulatory and respiratory systems and general symptoms and signs were 7%, 9.7%, 3.5% and 31.3%, respectively. Causality results linked 14.3% of symptoms to Falcimon® (Artesunate-Amodiaquine) with certainty. Effects were classified as mild and severe in 69.1% and 7.3% of cases respectively while 23.6% were serious. All patients with serious ADEs were hospitalized. One death was reported in a patient who had taken 24 pills at once.

**Conclusion:**

These results confirm the need to develop and implement pharmacovigilance systems in malaria endemic countries in order to monitor the safety of anti-malarial treatments.

## Background

Early diagnosis and prompt treatment with an effective anti-malarial drug remains a priority for the National Malaria Control Programme (NMCP) in Senegal. This strategy was threatened by the spread of chloroquine resistance and the NMCP adopted artemisinin-based combination therapy (ACT) in line with WHO treatment guidelines. In Senegal, two combinations are recommended as first-line treatment for uncomplicated malaria cases: artesunate-amodiaquine (AS-AQ) and artemether-lumefantrine (AL). In 2006, with the financial support of the Global Fund against AIDS, Tuberculosis and Malaria, the AS-AQ combination was scaled up in the Senegalese health care system [1].

ACT is based on a combination of drugs some of which have been recently marketed. Safety profile data for their long-term use in the general population in sub-Saharan Africa are scarce. Several studies have reported safe use of ACT, particularly the AS-AQ combination [2], but there is little data outside of clinical trials. Post-market safety data are important and must be collected and analysed in a standard way, considering that patients in endemic areas are likely to receive repeated anti-malarial treatment in the course of a year [3]. Unfortunately, in developing countries the incidence of adverse events in the general population is under-recognized [4], because the post-marketing drug surveillance systems are barely functional in most of the endemic countries, which explains why these countries have a low level of case reporting [5]. In most of these countries, lack of expertise and resources present barriers to a functional pharmacovigilance system. This phenomenon was reported in a recent study which stressed the lack of systematic and consistent inclusion of pharmacovigilance activities and requested funding support in proposals and in-country plans [6]. In the same study, a lack of consistency across proposals in what was defined and reported as pharmacovigilance demonstrated the need for stronger efforts to advance the role and effectiveness of pharmacovigilance activities to support malaria control and prevention programmes.

In Senegal, a national pharmacovigilance system based at the National Centre of Pharmacovigilance (“Centre Antipoison”) was created in 1998, but it was not scaled up and therefore the reporting was very poor until recently. Since 2009 Senegal has been a member of the WHO Collaborative Programme for International Drug Monitoring, based at the Uppsala Monitoring Centre, Sweden. Because pharmacovigilance should be an essential component for all public health programmes that use medicines, there was need to develop a pharmacovigilance system for antimalarials and in particular for ACT as part of the introduction plan of these new drugs. This brought together the competencies of the Ministry of health, the National Centre of Pharmacovigilance, academic and private sectors and partners such as WHO, UNICEF, USAID and civil society organisations under the coordination of the NMCP to develop and implement a dedicated safety monitoring system of antimalarial drugs in all the public health services in Senegal in 2007.

This paper reports Adverse Drug Events (ADEs) collected in health facilities after anti-malarial treatment between January 2007 and December 2009.

## Methods

### Study setting

Senegal is a West African country, with a population of approximately 12,141,673 inhabitants (2010). Malaria is endemic with a high transmission season between July and November. The entire country’s population is at risk of malaria.

Malaria case management is integrated into primary health services. Health services are provided nationwide through 75 health districts, which cover 78 district hospitals and 1,018 health posts. Implementation of the pharmacovigilance system involves all public health facilities. Between April 2006 and June 2009, artesunate + amodiaquine (AS + AQ) in a co-blister was used to treat uncomplicated malaria. In July 2009, co-blisters were replaced by fixed dose artesunate-amodiaquine (AS-AQ fixed dose). Drugs cost 0.6 US$ per course for adults and 0.3 US$ per course for children during the period covered by the study.

The treatment was administered once a day for three days using the following drugs:

Between April 2006 and June 2009: AS + AQ in co-blistered or Falcimon® (Cipla, India). It contains 50 mg of AS per tablet and 153 mg AQ base per tablet. The recommended doses were 4 mg/kg/day for AS and 10 mg/kg/day for AQ. The product was available in three formulations: three AS tablets and three AQ tablets for children 0–6 years, six AS tablets and six AQ tablets for children 6–13 years and 12 AS tablets and 12 AQ tablets for adults ≥ 14 years.

From July to December 2009: AS-AQ fixed-dose co-formulation or ASAQ Winthrop® (Sanofi-Aventis). There were four different formulations: AS 25 mg and AQ base 67,5 mg tablets for children 2–11 months; AS 50 mg and AQ base 135 mg tablets for children 1–5 years; and AS 100 mg and AQ base 270 mg tablets for children 6–13 years (three tablets) and for adults ≥ 14 years (six tablets).

This study was conducted countrywide and patients have been reported by public health facilities located in different regions in Senegal. All patients who received partial or full treatment of ACT and who presented ADEs which had been reported by health providers, were enrolled in the study.

### Study design

This study is a retrospective analysis of ADEs cases related to ACT as self-reported by patients at health facilities during a period of three years.

### Data collection and analysis

Data were collected by health workers using the standard ADEs reporting form developed by the Ministry of Health. Collected data were:(i) the patient (age, sex, identification number, antecedents, risk factor), (ii) all the drugs used by the patients (names, dose, dosage form, route, start/stop dates), (iii) the adverse reactions presented by the patient (date of onset, symptoms and signs, evolution), (iv) and the reporter (name, address, telephone, email).

When an ADE was diagnosed, the health worker filled the standard form and sent it out to the NMCP focal point for case management and pharmacovigilance through the District Health Officer. All forms were checked and validated, including telephoning the reporter if necessary. In all cases a letter was sent to the reporter to acknowledge receipt of the form.

After validation the forms were sent to the National Centre of Pharmacovigilance (*Centre Antipoison)* for causality analysis. Afterwards all reported cases and causality results were recorded in the NMCP pharmacovigilance database using Microsoft® Excel programme.

### The seriousness of the ADEs

Intensity of events was defined using the Moroccan pharmacovigilance centre grades, which identifies three grades of intensity: serious, severe and mild [7]. *Serious*: a serious adverse event was defined as one that was fatal, life-threatening, caused or prolonged hospital admission, caused persistent incapacity or disability, or caused congenital anomaly. *Severe*: ADE not serious, but drugs must be discontinued and an additional treatment initiated. *Mild*: minor symptoms, neither serious nor severe

### Causality

This is the relationship between the ADEs and the ACT. Causality was determined using the WHO method. The WHO causality categories benefit from long and extensive use and have the advantage of being internationally agreed and easy to use. The WHO method has six causality categories [8]. Causality assessment was performed on a daily basis by a team which includes a medical doctor, a pharmacist and a pharmacologist. Each quarter the results are reviewed and endorsed by the pharmacovigilance technical committee. An annual report is prepared and presented by the technical committee to the National Pharmacovigilance Advisory Committee, which involves all stakeholders.

The WHO causality categories are: (1) Certain: clinical event occurring in a plausible time relationship to drug administration, which cannot be explained by concurrent disease or other drugs of chemicals. The response to withdrawal of the drugs (dechallenge) should be clinically plausible. Finally, the event must be definitive pharmacologically or phenomenologically, using a satisfactory rechallenge procedure if necessary. (2) Probably/Likely: clinical event with a reasonable time sequence to administration of the drug; unlikely to be attributed to concurrent disease or other drugs or chemicals, which follows a clinically reasonable response on withdrawal (dechallenge). Rechallenge information is not required to fulfil this definition. (3) Possible: clinical event, with a reasonable time sequence to administrations of the drug, but which could also be explained by concurrent disease or other drugs or chemicals. Information on drug withdrawal may be lacking or unclear. (4) Unlikely: Clinical event with a temporal relationship to drug administration which makes a causal relationship improbable, and in which other drugs, chemicals or underlying disease provide plausible explanations. (5) Conditional/Unclassified: Clinical event, reported as an adverse reaction, about which more data is essential for a proper assessment, or the additional data is under examination. (6) Unassessable/Unclassifiable: a report suggesting an adverse reaction which cannot be judged because information is insufficient or contradictory, and which cannot be supplemented or verified.

Data analysis was done using Stata 11. Age was treated as a categorical variable using strata. In addition age and appearance of ADEs were summarized as means. Other socio-demographic variables and safety indicators were presented as frequencies and percentages. Incidence of ADEs was calculated using the number of treatment distributed per year. Lastly signs and symptoms have been classified on the basis of the International Statistical Classification of Diseases and Related Health Problems, 10th Revision (ICD-10) [9].

### Ethical considerations

This intervention was administered by the Ministry of Health and included in the routine work of health workers according to its malaria policy. Institutional Review Board clearance was not required.

## Results

### Socio-demographic characteristics

Between 1st January 2007 and 31st December 2009, a total of 123 patients with ADEs related to ACT were reported. The mean age of patients was 25.9 years (range 6 months-93 years) with a F/M sex ratio of 1.12 (Table 1). Sixty seven percent of the notifications were gathered from public health posts while district hospitals reported 30.1% of the ADEs. Only 2.4% of ADEs were collected from private health facilities. Nurses represented 86% of reporters while physicians and midwives represented 13% and 1% respectively.

**Table 1:** Distribution of cases according to age groups

**Ages (years)**	**N**	**%**
Under 5	11	9%
5-13	18	14.6%
14-25	39	31.7%
26-35	21	17%
36-45	11	9%
46-55	15	12.2%
Over 55	5	4%
Not specified	3	2.4%
Total	123	100%

### Reporting rate

There was on average 10 reported ADEs per 100 000 treatments distributed per year and an increase in the incidence of reported events between 2007 and 2009 according to consumed treatments (Table 2).

**Table 2:** Incidence of reported ADEs

**Year**	**ADE cases**	**ACT treatments distributed**	**Incidence of ADEs (per 100000 treatments)**
2007	64	990341	6.5
2008	34	338335	10
2009	25	184170	13.6

### Symptoms and signs

In total 227 signs and symptoms were collected among 123 patients. This represents on average two symptoms per patient. Thirty six per cent of patients present only one symptom such as nausea, vomiting, diarrhoea, pruritus and urticaria. The adverse events had appeared within a mean of 1.6 days (range 0 to 14 days) after the beginning of therapy while the duration of ADEs varied from less than 24 hours to 32 days. The events were reported following the use of three different combination therapies: AS + AQ co-blister, AS-AQ fixed dose and artesunate-mefloquine (AS-MFQ). Eighty five percent of the notifications were related to the AS + AQ coblister, while 14% were related to AS-AQ fixed dose and one reported case was related to AS-MFQ.

Gastrointestinal disorders represented 46.7% of symptoms and signs reported, followed by general symptoms and signs (31.3%). Disorders of the skin and cutaneous system, nervous system and circulatory and respiratory systems represented 9.7%, 7% and 3.5% of the effects respectively (Table 3).

**Table 3:** Classification of symptoms and signs

**Systems**	**Symptoms and signs**	**Frequency**
Digestive system and abdomen N* = 106 46.7%	Nausea and vomiting	59
	Abdominal pains	27
	Diarrhea	20
**Nervous system symptoms N = 16 7%**	Extrapyramidal and movement disorders	6
	Tremor	1
	Stiff neck	1
	Insomnia	5
	Somnolence	2
	Confusion	1
**Skin and subcutaneous system N = 22 9.7%**	Pruritus	14
	Urticaria	3
	Eruptions	3
	Rash	2
**Circulatory and respiratory system N = 8 3.5%**	Hiccough	2
	Dyspnoea	3
	Low blood pressure	3
**General symptoms and signs N = 71 31.3%**	Fever	4
	Headache	7
	Malaise and Fatigue	17
	Syncope	2
	Oedema	2
	Dry mouth	1
	Anorexia	3
	Tinnitus	6
	Profuse sweats	1
	Vertigo	28
**Others N = 4 1.8%**	Visual disorders	2
	Myalgia	1
	Fixed stare	1

*N = number of reaction terms and the percentage is of a total of 227 terms.

### Seriousness

ADEs were classified as mild in 69.1% of cases and 7.3% severe, while 23.6% of ADEs were serious. The percentage of serious effects was about two times higher with the AS-AQ co-blister than the fixed dose (25.7% vs. 11.7%). All the serious ADEs were hospitalized and there was one death due to misuse of drug. The patient was a 42-year old man who had taken all the 24 tablets of AS-AQ at once together with paracetamol and ciprofloxacin tablets. On the same day he overdosed, he had presented with movement disorders, vertigo, dyspnoea and hiccough and was admitted in a rural district hospital before he died.

### Causality

Using the WHO Causality criteria, 14.3% of ADEs related to AS-AQ co-blister treatment were classified as Certain. The main symptoms were nausea and vomiting, abdominal pains, diarrhoea, extra pyramidal and movement disorders, pruritus, urticaria, dyspnoea and hiccough. 22.8% and 37.2% of ADEs related to this combination were classified as Probable and Possible respectively. Twenty-seven reports were classified as Unlikely, Conditional/Unclassified or Unassessable/Unclassifiable. None of the events linked to use of the AS-AQ fixed dose were classified as Certain. However 23.5% of cases were classified as Probable and 53% as Possible. The main effects reported were nausea and vomiting, pruritus, vertigo, fatigue, tinnitus and confusion. Four reports were classified as Unlikely or Unassessable/Unclassifiable. One ADE related to AS-MFQ was reported. The patient presented with pruritus which was classified as Probably linked to this drug.

## Discussion

With the wide use of ACT in endemic areas, safety monitoring of anti-malarial drugs is critical. This study serves to reinforce previous reports advocating for increased pharmacovigilance on adverse drug events of artemisinin-based combination therapy, particularly in Africa [10-12]. While there exist several pre-clinical safety profile reports on different forms of ACT, pharmacovigilance-based reports on these combinations are scarce particularly in sub-Saharan Africa, a major market for these drugs.

Most of the ADEs were reported by nurses in peripheral health posts. Because routine spontaneous reporting relies on vigilance of health workers, all the ADEs may not be fully reported, this could lead to a low reporting rate. Even though data were limited and based on passive reporting, the causality classifications support a link between the adverse events and administration of ACT. Adverse drug events are different from adverse drug reactions. While an adverse event is any undesirable medical occurrence that develops after administration of a drug, regardless of the suspected relationship between the drug product and the event, to classify the event as an adverse drug reaction, a causal relationship must be established [3].

One difficulty in this study was distinguishing between adverse events and common symptoms of malaria [2]. Indeed symptoms presented by patients were not documented before treatment in the standard form to make correct difference between malaria signs and ADEs symptoms. But this was considered during the causality analysis as the WHO method takes into account the fact that the events could also be explained by concurrent disease or other drugs or chemicals. It was also difficult to establish whether patients were admitted in hospital because of the seriousness of the symptoms or because health workers wanted to avoid evolution to complicated malaria by administering parenteral treatment to prevent deterioration in the patient’s condition [13].

The apparent incidence of ADEs is far less than the results of a clinical trial conducted in Senegal. That is undoubtedly a reflection of the nature of the reporting scheme, which relies on busy nurses and other health professionals to report adverse events they suspect are related to the anti-malarial medications. In addition, no biological effects have been reported because all the ADEs were collected from peripheral health facilities where there is no laboratory service [14]. The frequency of gastro-intestinal symptoms related to AS-AQ has been reported by others studies [2,15]. All the serious events were linked to artesunate-amodiaquine, particularly with the co-blister combination, which has been used for a longer period of time in comparison to other combinations. All patients recovered. The only case of death reported was due to a misuse of the drug.

This study’s findings confirm the relative safety of use of ACT as reported by previous studies [2,14,15]. Most of the ADEs were expected as they are mentioned in the Summary of Products Characteristics (SPC). A few general symptoms and signs and extra pyramidal reactions were unexpected and, therefore, not found in the SPC. Our series reported 6 cases of extrapyramidal reactions among 4 children and 2 adults. A recent publication supports an association of the use of AS-AQ combination therapy with acute extrapyramidal reactions, which occurred with recommended, and in some instances reduced, daily doses [16].

## Conclusion

Although endemic countries may have inadequate infrastructure and limited resources for implementing pharmacovigilance, drug safety monitoring mechanisms are imperative. This study demonstrates the feasibility and the need to set up a safety monitoring system for anti-malarial drugs within a national pharmacovigilance system. The latter should be scaled up in health facilities and hospitals and involve all health workers to improve the notification of adverse drug events of ACT. Even though the safety of ACT is established, post-marketing safety monitoring of ADEs is critical and should be developed and implemented in endemic areas. Furthermore because many endemic countries are scaling up malaria case management at community level, and given that people living in malaria areas can receive more than one course of treatment a year, the safety monitoring system of ACT should not only involve health facilities but also be extended at community level.

## Competing interests

The authors declare that they have no competing interests.

## Authors’ contributions

ST coordinated this study and devised the study objectives and design. ST and JLN designed the methodology, did the analysis and wrote the manuscript. ID, FBF, MLD, MBD, MN carried out the planning, development of tools, training, implementation and monitoring during the study. NED assisted with data collection, data cleaning and analysis. BF assisted with training and supervision. PG assisted in writing the first draft and in revising the manuscript. OG and MT oversaw this study. All authors read and approved the manuscript.
